# PlantFuncSSR: Integrating First and Next Generation Transcriptomics for Mining of SSR-Functional Domains Markers

**DOI:** 10.3389/fpls.2016.00878

**Published:** 2016-06-27

**Authors:** Gaurav Sablok, Antonio J. Pérez-Pulido, Thac Do, Tan Y. Seong, Carlos S. Casimiro-Soriguer, Nicola La Porta, Peter J. Ralph, Andrea Squartini, Antonio Muñoz-Merida, Jennifer A. Harikrishna

**Affiliations:** ^1^Plant Functional Biology and Climate Change Cluster (C3), University of Technology, SydneyNSW, Australia; ^2^Centro Andaluz de Biología del Desarrollo (CABD-CSIC), Universidad Pablo de OlavideSevilla, Spain; ^3^Centre for Research in Biotechnology for Agriculture and Institute of Biological Sciences, Faculty of Science, University of MalayaKuala Lumpur, Malaysia; ^4^Department of Sustainable Agro-Ecosystems and Bioresources, Research and Innovation Centre, Fondazione Edmund MachTrento, Italy; ^5^MountFOR Project Centre, European Forest InstituteTrento, Italy; ^6^Consiglio Nazionale delle Ricerche, Istituto per la Valorizzazione del Legno e delle Specie ArboreeFlorence, Italy; ^7^Department of Agronomy, Food, Natural Resources, Animals and Environment, University of PadovaPadova, Italy; ^8^CIBIO Research Centre in Biodiversity and Genetic Resources, InBIO, Universidade do PortoVila do Conde, Portugal

**Keywords:** short tandem repeats (STRs), NGS, gene ontology (GO), inter-pro, functional domains markers

## Abstract

Analysis of repetitive DNA sequence content and divergence among the repetitive functional classes is a well-accepted approach for estimation of inter- and intra-generic differences in plant genomes. Among these elements, microsatellites, or Simple Sequence Repeats (SSRs), have been widely demonstrated as powerful genetic markers for species and varieties discrimination. We present PlantFuncSSRs platform having more than 364 plant species with more than 2 million functional SSRs. They are provided with detailed annotations for easy functional browsing of SSRs and with information on primer pairs and associated functional domains. PlantFuncSSRs can be leveraged to identify functional-based genic variability among the species of interest, which might be of particular interest in developing functional markers in plants. This comprehensive on-line portal unifies mining of SSRs from first and next generation sequencing datasets, corresponding primer pairs and associated in-depth functional annotation such as gene ontology annotation, gene interactions and its identification from reference protein databases. PlantFuncSSRs is freely accessible at: http://www.bioinfocabd.upo.es/plantssr.

## Introduction

Identification of repetitive patterns in genomic DNA has proved to be a powerful approach to reveal diversity and to discriminate plant populations and individuals within species. Microsatellites or Simple Sequence Repeats (SSRs) formed as a result of the strand-slippage mechanism (Schlötterer and Harr, 2001) have been used widely as functional genetic markers (Studer et al., 2010), for testing genetic fidelity, genetic variability (Rahman and Rajora, 2002; Schellenbaum et al., 2008) and for population genetic studies (Sim et al., 2009). However, the previously described approaches such as by screening the small insert genomic DNA libraries (Shokeen et al., 2007) are time consuming and not so cost effective. Furthermore, SSRs identified by such approaches have no certainty of association to the functional domains. Leveraging the computational advances, *in silico* mining approaches using transcriptomics have filled a major gap in the development of these functional classes of markers (Sablok and Shekhawat, 2008; Sablok et al., 2011), which could be potentially used for developing the markers harboring the functional domains for marker assisted gene selection, genotyping, and anchoring quantitative trait localization (QTL; Parida et al., 2010; Kujur et al., 2013) mainly due to the associative nature of the mined SSRs to the coding region variations and the associated functional variations.

Recently, several SSRs have been linked to putative functional domains; classifying them into a new class of functional markers called simple sequence repeats functional domains markers (SSR-FDMs) in model and non-model species (Yu et al., 2010; Bhattacharyya et al., 2014). Realizing the wide importance of SSRs, several online repositories and data mining tools have been developed to address the need for on-line mining of these markers in case of nuclear genomes such as PlantMarkers (Rudd et al., 2005), SSR Biome and SSR taxonomy (Jewell et al., 2006), UgMicroSatDb (Aishwarya and Sharma, 2008), MoccaDB (Plechakova et al., 2009), CicArMiSatDB (Doddamani et al., 2014), and for Coffee expressed sequence tags (ESTs) (Poncet et al., 2006) to assist the mining of the SSRs. However, there are some limitations to the previously developed tools that have restricted, in particular, the possibility to make comparisons across different datasets from different species as they either lack integration of the browsing platform with unified annotations or they are oriented toward specific species such as CicArMiSatDB (Doddamani et al., 2014), and FmMDb (B et al., 2013). In case of organelle genomes, we previously established ChloroMitoSSRDB (Sablok et al., 2013) and ChloroMitoSSRDB 2.00 (Sablok et al., 2015) to provide the large-scale access to the organelle derived markers.

Next generation sequencing (NGS) provides a cost-efficient way of transcript identification and facilitates the development of transcript based SSRs markers for model and non-model species, which has resulted in rapid increases in the data made available online. However, much of this data is scattered across numerous websites and has not been mined or annotated for the identification of functional SSRs. Recently, there have been some efforts to consolidate such data for example TropiTree^1^ is a repository displaying the mined SSRs from NGS transcript assemblies for 24 tropical plants (Russell et al., 2014). Taking into account the limitations mentioned, we were motivated to develop PlantFuncSSRs, available at http://www.bioinfocabd.upo.es/plantssr, which is a unified functional SSRs portal displaying mined functional SSRs from 274 ESTs based transcript assemblies, and more than 100 NGS transcripts assemblies. PlantFuncSSRs also provides detailed primer pair information, functional annotations, and putative homologs to the transcript assemblies in Uniprot and curated SSR-FDMs in a single unified platform. We believe that the availability of the above resource will aid the rapid development of functional SSRs in non-model plant species.

## Materials and Methods

### Data Resources for PlantFuncSSRs

To integrate previously published plant EST data, all Putative Unique Transcripts (PUT) representing 273 transcript assemblies were downloaded from PlantGDB (Version release 187) available from http://www.plantgdb.org/ (Dong et al., 2004). Additionally, version control 74 NGS transcriptomes available at PhytoMetaSync^2^ (Facchini et al., 2012; Xiao et al., 2013), 14 medicinal plant transcriptomes available from medicinal plant genomics resource (MPGR)^3^ (Góngora-Castillo et al., 2012; Góngora-Castillo and Buell, 2013) and 3 *Brachypodium sylvaticum* transcriptomes available from http://jaiswallab.cgrb.oregonstate.edu/genomics (Fox et al., 2013) were downloaded, representing a total of 364 plant species.

### SSRs Identification and Functional Assignments

For systematic identification of SSR, all the transcripts (ESTs as well as NGS) assemblies were first scanned for the presence of the homopolymer errors and sequence ambiguity was removed using the est_trimmer tool available at: http://pgrc.ipk-gatersleben.de/misa/download/est_trimmer.pl with the following settings: -amb = 2.50 -tr5 = *T*, 5.50 -tr3 = *A*, 5.50. Following the transcript ambiguity removal and trimming of the homopolymer runs, MISA (MIcroSAtellite identification tool) (Thiel et al., 2003) was deployed to identify the microsatellites. In the present version of the PlantFuncSSRs, we classified microsatellites as repetitive stretches of motifs of a minimum and 12-mer repetitive stretch as mono-, 6-mer repetitive stretches as di, 4-mer repetitive stretches of tri- and tetra-, and a minimum of 3-mer repetitive stretch as penta- and hexa-nucleotide. Additionally, the identified SSRs have been classified into perfect and compound repeats, with compound repeats interrupted by a minimum of 100 bp as previously described (Victoria et al., 2011). Primer pairs were designed for all of the identified SSRs using primer3 available from primer3.sourceforge.net (Untergasser et al., 2012) using the settings as described in MISA (Thiel et al., 2003).

Following SSRs identification, in-depth functional annotation of the identified SSRs was carried out using the standalone annotator Sma3s (Muñoz-Mérida et al., 2014), which uses the plant taxonomic division set in the Uniprot database^4^, including both Swiss-Prot and TrEMBL sections to enrich the final annotation. The annotations gave the found Gene Ontology (GO) terms which were subsequently linked to their GO_SLIM terms using the plant GO slim available from www.geneontology.org, in order to simplify the GO terms and allow cross-comparison. In this way, each SSRs sequence was identified with the more probable gene name and description, as well as both GO terms from the existing three categories and Swiss-Prot keywords, all of them for cataloging the SSRs and assigning functional domains. The IntAct annotations and Interactions were cross-linked using the IntAct resources available from EBI at: http://www.ebi.ac.uk/intact/. The functional SSRs annotation also includes putative InterPro domains (Quevillon et al., 2005, pathways from UniProt to have more details of the involved biological processes. PlantFuncSSRs presents only those SSRs, which have functional annotations appended to them and are thus termed as SSR-functional markers.

## Results and Discussion

### PlantFuncSSRs Architecture and Visualization

**FIGURE 1:**
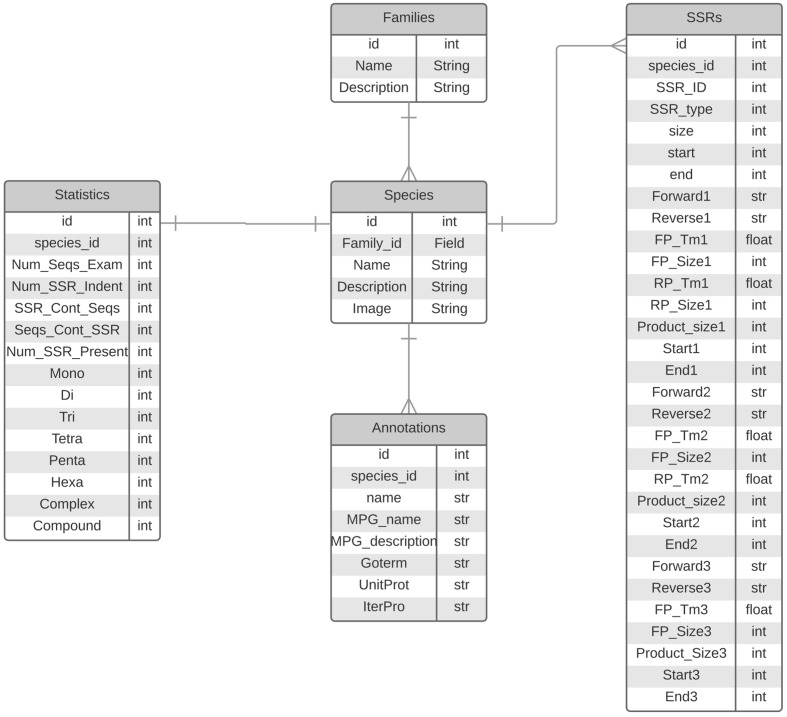
**Entry–Entity relationship diagram of PlantFuncSSRs**.

**FIGURE 2:**
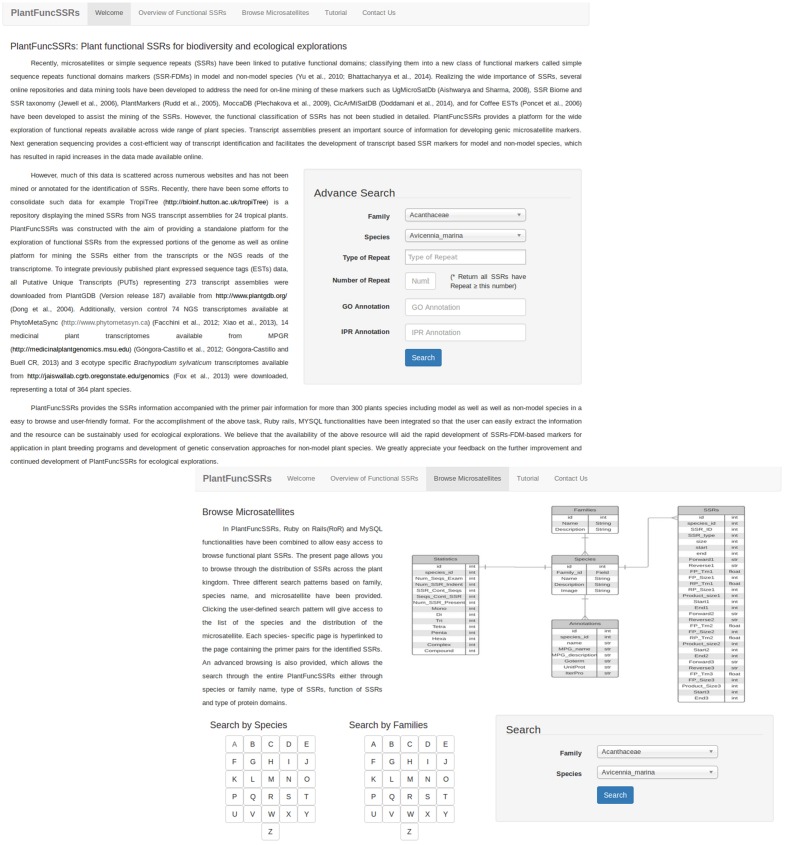
**PlantFuncSSRs: Schematic view of the PlantFuncSSRs and the browsing options implemented in PlantFuncSSRs.** The structure of PlantFuncSSRs allows for the browsing of the functional SSRs either according to the species or according to the family.

**FIGURE 3:**
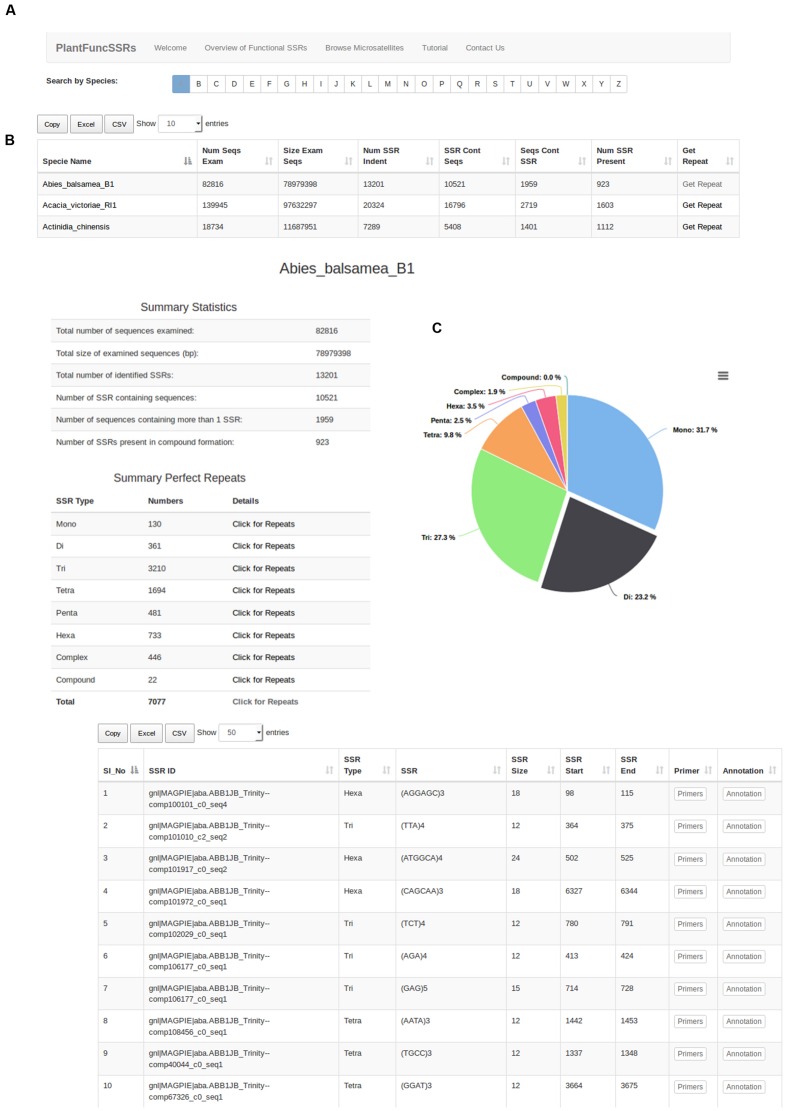
**Alphabet sorting of the species names and search patterns **(A)**; Species specific page showing the information on the identified Simple Sequence Repeats (SSRs) and also the functional SSRs.** “Click for repeats” pages are directly hyperlinked to the functional SSRs **(B)**; Weblayout describing the functional repeats identified in the respective plant species with information on type of repeat, classification of repeat, size, motif, start, and end coordinates and associated primers and functional annotation **(C)**.

Expressed sequence tags and NGS based Transcriptome reconstruction represent the functional portion of the genome and have been widely used as resources to mine and develop functional markers. Developing an efficient browsing system for the mining of repeats is an important task, as this can be widely applied to a wide range of on-going plant breeding and crop improvement research. To develop an efficient browsing system, PlantFuncSSRs architecture has been developed using Ruby Rails and MySQL, which provides faster integration and query based searches to the users. The current version of the PlantFuncSSRs presents more than 2 million SSRs and SSR-FDMs from 364 species for easy access and browsing of transcript derived plant SSRs across the plant kingdom (**Table 1**). These species are ranging from important crops to wild species, from mono- to di-cots, from annual to polyannual and wood species. Integration of visualization features with the rapid mining of the data is a key central feature that has been implemented in the PlantFuncSSRs. A schema of the database architecture in the form of entity-relationship is given in **Figure 1.** For the visualization of the SSRs and the associated information, several hierarchal levels of classified information have been inter-linked in PlantFuncSSRs (**Figure 2**). The front-end portal is user-friendly and allows the end-users to search SSRs as “species-wise”, “family wise”, or “advanced search menu” (**Figure 2**). A quick search implementation pattern displays the embedded species information in quick select “*species*” and “*families*”, which are hyperlinked pages to the respective species and provide a quick view of the functional SSRs present in each species. **Figure 3** shows the webpage browsing of PlantFuncSSRs with detailed classification of the identified SSRs for user-selected species of interest. Alphabetical classification of the species provides an additional advantage for the users to quickly look for their species of interest (**Figure 3**).

**Table 1:** Table describing the classified repeats types and embedded functional categories in PlantFuncSSRs.

Type of SSRs	Number of SSRs
P1 SSRs-FDMs	221008
P2 SSRs-FDMs	200702
P3 SSRs-FDMs	1067949
P4 SSRs-FDMs	358245
P5 SSRs-FDMs	102593
P6 SSRs-FDMs	142452
Compound SSRs-FDMs (C and C^∗^)	292472
Functionally embedded SSRs annotations	
Gene names	2278574
Descriptions	2332906
Gene ontologies	1986736
Uniprot (keywords)	2122976
InterPro domains	2172553


**FIGURE 4:**
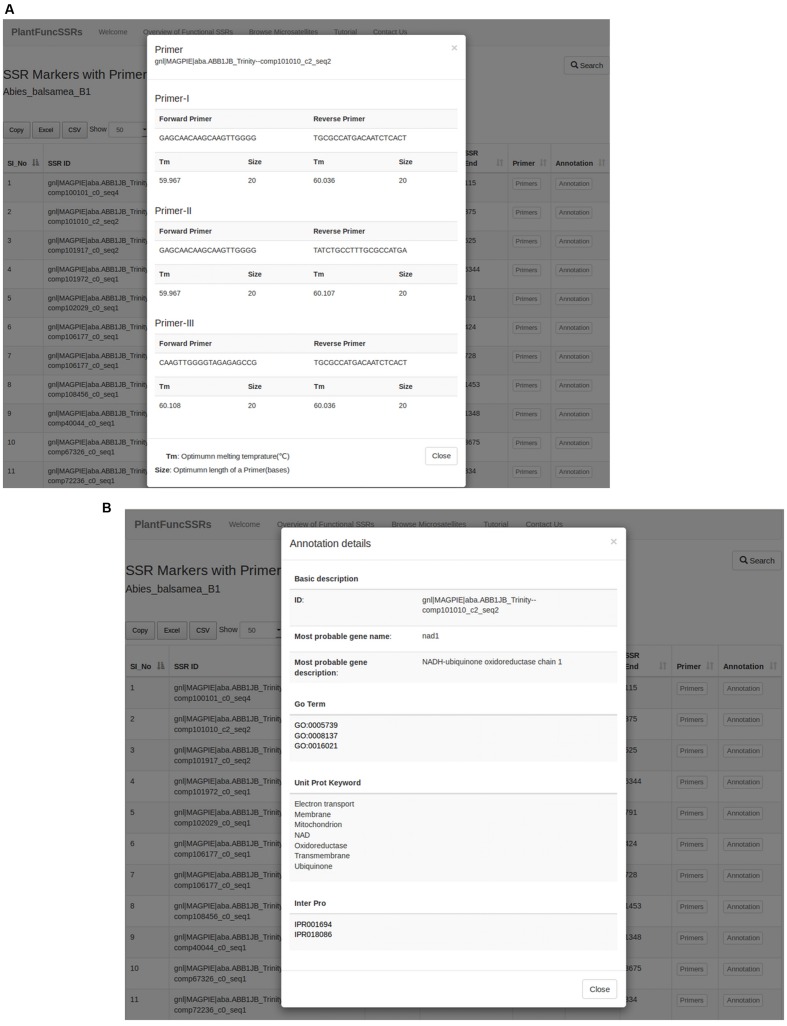
**Pop-up Primer display window for the user selected functional SSRs **(A)**; Pop-up window showing high throughput functional annotation for the user selected functional SSRs **(B)****.

Each record in the species displays the *Species_Name, Num_Seqs_Exam, Size_Exam_Seqs, Num_SSR_Ident, SSR_Cont_Seqs, Seqs_Cont_SSR*, and *Num_SSR_Present* providing summarized information on the number of the identified SSRs for that particular species of interest lined to the primer pair information and high throughput functional annotation (**Figure 3**). In PlantFuncSSRs, each species page has been hyperlinked to the corresponding repeat information pages that present detailed information on several statistics such as total number of sequences examined, total size of examined sequences (bp), total number of identified SSRs, number of SSR containing sequences, number of sequences containing more than one SSR and compound SSRs (**Figure 3**). In addition, to this summary information, each species classified page also details the types and distribution of the repeats in tabular format, which can be sorted “*on the fly*”. An integral part of PlantFuncSSRs is to describe the associated primer pair information for each species to facilitate the development of functional SSRs for diversity analysis. To augment such capacity, each functional SSR has been associated with primer pages and detailed functional annotations, which describes the set of the “ready to use” primers for the functional validation of the corresponding SSRs (**Figure 4**).

### Functional SSRs and Functional Importance of PlantFuncSSRs

Microsatellites (SSRs) have been shown to be regulators of a number of plant genes demonstrating their importance as key players in regulating plant function (Faville et al., 2004). FuncPlantSSRs offers a wide variety of functional annotations for the identified SSRs such as GO terms, GO slim categories, pathways, descriptions to identify the sequences and comparing with putative homologues, and motif and domain modules to offer the domain architecture for the sequences. Recently, increasing interest toward the functional linkage of the markers to the domain association and function can be seen from several recent reports in plants such as *Ocimum basilicum* (Gupta et al., 2010), *Seasmum indicum* (Bhattacharyya et al., 2014), *Elaeis guineensis* (Tranbarger et al., 2012), and *Camellia sinensis* (Sahu et al., 2012) suggesting the role of the functional SSRs as important markers for developing the functional genic approaches for marker enrichment in plants. Nonetheless, established reports of the functional association of the repeats with the catalytic domains (Parida et al., 2010; Yu et al., 2010) has been widely developed. For quick advanced searches, PlantFuncSSRs offer several functionalities, such as searches customized and optimized on various hierarchal levels i.e., Family, Species, Type of Repeat, Number of Repeat, Functional annotation, GO annotation, and IPR annotations (**Figure 2**). Availability of the curated information provides end users with the flexibility to narrow their searches to functional SSRs linked to specific categories, motif types or functional annotations. Taking into account the vast amount of the species coverage and associated functional SSRs present in the PlantFuncSSRs, we believe that the PlantFuncSSRs provides access to the most comprehensive catalog available for the functional SSRs from plant transcriptomes.

## Conclusion

In the present version of the PlantFuncSSRs, we bring together under a unified portal the mining of the SSRs from the publically available first and second generation datasets. PlantFunctSSRs has been designed with an aim to serve as a stand-alone single access platform for the analysis of functional SSRs from first and NGS datasets for a large number of sequenced plant transcriptomes. In addition to providing the most comprehensive available resource for exploring and validating plant functional SSRs, the built in annotation platform will allow the users to have wide access to the functional relevance of the validated SSRs thus provides a valuable functional SSRs resource to support plant diversity, population and functional marker research.

## Author Contributions

GS conceived and designed the research, identified SSRs and linked the SSRs to functions, AP and AM-M provided the annotation, TD build the database and the web-interface, TYS helped in the data integration, CSCS hosted the database, GS wrote the manuscript, NP, AS, PR, and JAH provided revisions. All authors have read and approved the manuscript.

## Conflict of Interest Statement

The authors declare that the research was conducted in the absence of any commercial or financial relationships that could be construed as a potential conflict of interest.

## Footnotes

1 http://bioinf.hutton.ac.uk/tropiTree

2 http://www.phytometasyn.ca

3 http://medicinalplantgenomics.msu.edu

4 http://www.uniprot.org
